# Resting echocardiographic parameters to detect patients with less symptomatic primary mitral regurgitation who require exercise stress echocardiography

**DOI:** 10.20407/fmj.2022-038

**Published:** 2023-08-28

**Authors:** Yuka Kawada, Akira Yamada, Shinji Jinno, Chihiro Nakashima, Naoki Hoshino, Sayano Ueda, Meiko Hoshino, Sayuri Yamabe, Kayoko Takada, Kunihiko Sugimoto, Hideo Izawa

**Affiliations:** 1 Department of Cardiology, Fujita Health University School of Medicine, Toyoake, Aichi, Japan; 2 Clinical Laboratory, Fujita Health University Hospital, Toyoake, Aichi, Japan; 3 Faculty of Nursing, Fujita Health University School of Health Sciences, Toyoake, Aichi, Japan

**Keywords:** Primary mitral regurgitation, Exercise stress echocardiography, Left atrial volume index

## Abstract

**Objectives:**

We aimed to identify which resting echocardiographic parameters can detect asymptomatic or mildly symptomatic patients with primary mitral regurgitation (MR) who require exercise stress echocardiography (ESE) to determine their suitability for surgery.

**Methods:**

We examined 56 consecutive patients with primary moderate/severe MR who underwent ergometer-based ESE. Patients who met the surgical indications at rest were excluded. Eligible patients were divided into Group I (pulmonary artery systolic pressure [PASP] during exercise >60 mmHg; n=11) and Group II (PASP during exercise ≤60 mmHg; n=30).

**Results:**

Forty-one patients were included. Group I was significantly older (65±12 vs. 54±14 years, P=0.042) and had significantly higher serum N-terminal pro-B-type natriuretic peptide concentrations than Group II (351±278 vs. 125±163 pg/mL, P=0.002). The univariate analysis demonstrated that peak E wave velocity (Group I vs. Group II: 125±45 vs. 101±24 cm/sec, P=0.050), left ventricular (LV) end-diastolic diameter index (32±4 vs. 30±3 mm/m^2^, P=0.035), and left atrial volume index (LAVI; 45±14 vs. 30±11 mL/m^2^, P=0.008) were predictors of increased PASP during exercise. In the multivariate analysis, resting LAVI best predicted exercise-induced pulmonary hypertension (hazard ratio 1.081 [95% confidence interval 1.009–1.158], P=0.028), with a cutoff value of 37 mL/m^2^.

**Conclusions:**

In asymptomatic or mildly symptomatic patients with primary moderate/severe MR, increased resting LAVI indicates the requirement for ESE, even without LV dilatation.

## Introduction

Mitral regurgitation (MR) is the most common valvular disorder worldwide, occurring in up to 10% of the general population.^1,2^ Moderate or severe MR is notably common, and its prevalence increases with age.^3^ MR is associated with a high mortality rate and frequent heart failure after diagnosis, even in those with normal left ventricular (LV) ejection fraction (LVEF) and a low rate of comorbidities.^1^ Despite these poor outcomes, some affected patients undergo mitral surgery, even in communities with readily available diagnosis and treatment methods.

**Figure 1:**
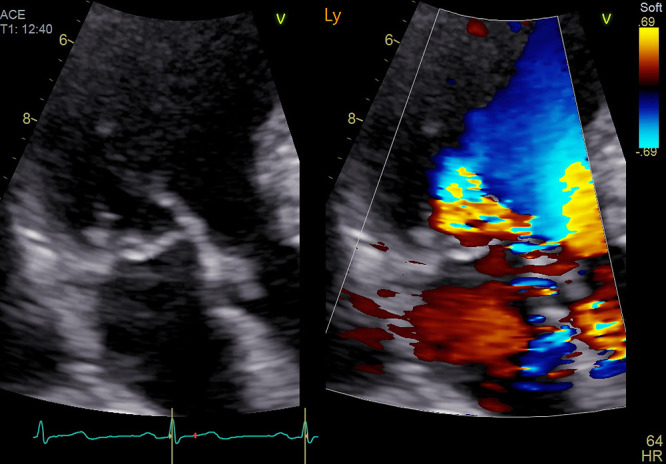
Representative case of primary severe MR caused by P2 prolapse MR, mitral regurgitation.

MR is generally underpinned by one of two mechanisms: primary MR (Figure 1), which is caused by valve degeneration, or secondary MR, which is caused by leaflet tethering without valve abnormalities, followed by LV and/or left atrial (LA) dilatation.

Performing surgical intervention for patients with primary MR at an appropriate time is important because mitral valve repair and a sufficient MR decrement contribute to an improvement in heart failure symptoms and a better prognosis.^4–6^ However, less than half of patients with moderate-to-severe or severe primary MR undergo surgery, and many are not referred for surgical consultation.^2^ One of the main reasons for this is the absence of symptoms, despite already having severe MR.

**Figure 2:**
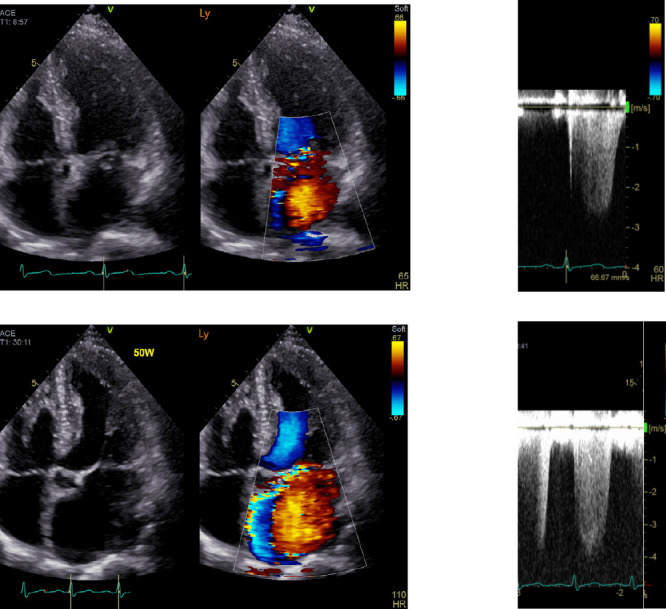
Representative case of exercise-induced PH Apical four-chamber image with color Doppler (upper left) and TR flow (upper right) at rest. Increased mitral regurgitation (lower left) and TRPG suggesting PH (lower right) during exercise. TR, tricuspid regurgitation; TRPG, tricuspid regurgitation pressure gradient; PH, pulmonary hypertension.

In the latest guidelines for MR,^7–9^ surgical treatment is generally recommended as the class I treatment for symptomatic patients with severe primary MR; being symptomatic is not so difficult to judge on surgical indication for MR.^10,11^ However, in asymptomatic patients with severe MR, decision-making is challenging, and complications, such as LV systolic dysfunction, atrial fibrillation (AF), and increased pulmonary artery pressure, can be the reasons for choosing surgical intervention. Furthermore, exercise stress echocardiography (ESE) is a strongly recommended option for patients classified as class IIa.^7,12^ ESE is also useful for possibly symptomatic patients with moderate MR. Although symptoms are subjective, clinically objective real-time parameters can be obtained by ESE. Exercise-induced pulmonary hypertension (PH), which is defined as a pulmonary artery systolic pressure (PASP) of >60 mmHg, is more common in patients with asymptomatic primary MR and is superior to resting PH for predicting symptom occurrence and the need for surgery during follow-up.^13^ Once exercise-induced PH is confirmed, earlier surgical intervention should be performed before resting symptoms become evident (Figure 2). This prompt intervention can lead to a better prognosis in these patients.

Recent advances in surgery for MR have emphasized the importance of appropriately selecting patients requiring earlier surgical intervention. However, the use of ESE differs between institutions, and not all patients who are eligible for ESE have the opportunity to undergo such a procedure because of limited accessibility, inadequate equipment, and lack of sonographers or cardiologists who are sufficiently skilled to perform ESE. The absence of a uniform protocol is another hindrance to the widespread use of ESE.

In this study, we aimed to determine the resting echocardiographic parameters that can identify asymptomatic or mildly symptomatic patients with primary moderate/severe MR who require ESE.

## Methods

### Study Subjects

We retrospectively enrolled 56 consecutive patients with moderate/severe primary MR (asymptomatic or mildly symptomatic) who underwent ergometer-based ESE at our institution between January 2011 and August 2021. The exclusion criteria were as follows: LVEF of <60%, LV end-systolic diameter (LVESD) of ≥40 mm, AF, and a resting PASP of >50 mmHg,^14^ as well as new appearance of LV wall motion abnormalities under stress.

### Echocardiography

Echocardiography was performed using the Philips iE33 (Amsterdam, the Netherlands) or GE Vivid E95 (Boston, US) system. The following echocardiographic parameters were assessed: LV end-diastolic diameter index (LVEDDI), LVESD index, LA diameter, LV end-diastolic volume index, LV end-systolic volume index, LVEF, maximum LA volume index (LAVI), and tricuspid regurgitation (TR) pressure gradient. All of these parameters were measured at rest and during exercise. LVEF was obtained using the biplane disc summation method. LV stroke volume was calculated by multiplying the LV outflow tract area by the LV outflow tract velocity time integral measured by pulsed wave Doppler. The mitral peak E wave velocity was also measured using pulsed wave Doppler. PASP was derived from the regurgitant jet of TR using the systolic trans-tricuspid pressure gradient, which was calculated using the modified Bernoulli equation with the addition of 3, 8, or 15 mmHg depending on the right atrial pressure.^15^ This pressure was defined by the diameter and respiratory fluctuations in inferior vena cava diameter.^16^ MR was considered primary if any one of the following findings was detected: flail leaflet, ruptured papillary muscle, severe retraction, or large perforation. MR severity was qualitatively assessed using the color flow jet area (large central jet with >50% of the left atrium or eccentric wall impinging the jet of variable size), with quantitative assessment as required by the latest guidelines.^8^

### Exercise Echocardiography

After the resting echocardiographic parameters had been measured, all patients performed a symptom-limited graded bicycle exercise test in the semi-supine position on a dedicated tilting exercise table. The initial workload of 25 W was maintained for 3 minutes; then, the workload was increased by 25 W every 3 minutes. Heart rate, blood pressure, oxygen saturation, and 12-lead electrocardiography were recorded every 3 minutes. Two-dimensional and Doppler echocardiographic images were procured throughout the test. The patients were classified into two groups: PASP of >60 mmHg (Group I) and PASP of ≤60 mmHg (Group II) at peak exercise. The two groups were then compared to detect their clinical characteristics at rest.^13,17^

### Statistical Analysis

The data are expressed as the mean±standard deviation or number (percentage), unless otherwise specified. Significant differences between the two groups were identified using the Student’s *t*-test or Wilcoxon’s rank-sum test, as appropriate. Various cutoff values for predicting an increased PASP of >60 mmHg during exercise were determined using the receiver operating characteristic (ROC) curve analysis. Furthermore, the ability of the variables to predict increased PASP during exercise was evaluated using the logistic regression analysis. All variables in the univariate analysis were included in the multivariate analysis. A P value of ≤0.050 was considered statistically significant. All statistical analyses were performed using JMP, version 15.1.0 (SAS Institute, Cary, US).

### Ethics Statements

The institution’s ethics committee approved the retrospective evaluation of the clinically acquired data (HM20-161). The study conformed to the provisions of the Declaration of Helsinki. The requirement for informed consent was waived due to the retrospective nature of the study.

## Results

This study included 41 patients, of which 11 (27%) were allocated to Group I and 30 (73%) were allocated to Group II. Group I was significantly older than Group II (65±12 vs. 54±14 years, P=0.042). Serum N-terminal pro-B-type natriuretic peptide (NT-proBNP) concentration (351±278 vs. 125±163 pg/mL, P=0.002), hypertension as a comorbidity (82% vs. 40%, P=0.018), and diuretic use (36% vs. 10%, P=0.047) were significantly higher/more frequent in Group I than in Group II. No other statistically significant differences in demographic or clinical data were identified between the two groups (Table 1).

In terms of the resting echocardiographic parameters, the univariate analysis showed that the peak E wave velocity, LVEDDI, and LAVI were significant predictors of increased PASP during exercise. These parameters were significantly higher in Group I than in Group II (peak E wave velocity: 125±45 vs. 101±24 cm/sec, P=0.050; LVEDDI: 32±4 vs. 30±3 mm/m^2^, P=0.035; LAVI: 45±14 vs. 30±11 mL/m^2^, P=0.008). No other statistically significant differences in resting or exercise echocardiography data were observed between the two groups (Table 2). In the multivariate analysis, LAVI was the only significant predictor of exercise-induced PH (Table 3).

The ROC curve analysis revealed that the cutoff value for resting LAVI to predict a PASP of >60 mmHg was 37 mL/m^2^ (area under the ROC curve, 0.81; sensitivity, 73%; specificity, 77%).

## Discussion

This study observed the following results with regard to patients with MR with exercise-induced PH: (i) they were older, with increased serum NT-proBNP concentrations; and (ii) with regard to echocardiographic parameters, they showed LA and LV enlargement, with a higher peak E wave velocity at rest. Resting LAVI was the strongest predictor of exercise-induced PH.

According to current guidelines, a resting PASP of >50 mmHg indicates the requirement for surgical intervention for MR.^7–9^ If the resting PASP is <50 mmHg in asymptomatic patients with moderate or severe MR, ESE is recommended to determine the next therapeutic step. An estimated PASP of >60 mmHg during ESE demonstrates the surgical indication as class IIb^7^ because a PASP of >56 mmHg during exercise is an accurate predictor of symptom occurrence and is associated with markedly lower 2-year symptom-free survival.^18^ A previous study reported that surgery for MR should be considered when the resting LAVI is >60 mL/m^2^ (class IIa).^8^ However, according to American Heart Association/American College of Cardiology guidelines for valvular heart disease management, LAVI is not included as a decision-making factor for patients with primary severe MR.^9^

Patients with worsening MR during ESE are in a state of chronic LA volume overload, leading to an increase in LAVI at rest. One study concluded that the measurement of LAVI should be included in the routine primary MR evaluation and the clinical decision-making process because LAVI has a powerful, incremental, and independent link to excess mortality, which can be partially alleviated by mitral surgery. During a 10-year follow-up, a LAVI of ≥40 mL/m^2^ showed the highest mortality rate.^19^ In the present study, 37 mL/m^2^ was the cutoff value for LAVI to predict exercise-induced PH. Therefore, even if the LAVI is slightly lower than 40 mL/m^2^ (i.e., 37 mL/m^2^), ESE should be considered to detect subclinical surgical indications for MR. This strategy would help to select patients with potential surgical indications at an earlier stage of progression of resting LAVI before reaching >60 mL/m^2^, in turn improving prognosis.^20^ Earlier surgery is associated with significant long-term reductions in cardiac mortality and cardiac events in asymptomatic patients with primary severe MR. These benefits are evident among patients aged ≥50 years.^21^ These findings emphasize the importance of identifying patients with primary MR who may be candidates for surgery.

In another report, the peak E wave velocity was an independent predictor of cardiovascular events in asymptomatic patients with primary MR with preserved LV function.^22^ However, in our study, the peak E wave velocity at rest insufficiently predicted an increase in PASP during exercise, which suggests a surgical indication as compared with resting LAVI, possibly because of differences in the patient background. The mean age of the patients in the previous study was similar to our study. However, the previous study only included patients with severe MR, and their LA size was more enlarged with a higher E wave velocity, indicating that the MR of the patients in that study was more advanced than in our study. In contrast, our study not only included patients with severe MR, but it also included patients with moderate MR. Our results show that resting LAVI is a more sensitive indicator of MR progression than the peak E wave velocity at an earlier stage of MR.

### Study Limitations

This study has some limitations that should be noted. First, this study was a single-center study with a relatively small sample size. Second, ESE requires experienced sonographers to record appropriate images in a limited period of time; therefore, it is possible that their level of skill might have influenced the echocardiographic measurements. Nevertheless, the image quality and measurements were double-checked by experienced cardiologists. Therefore, differences in the experience of the sonographers likely would not have affected the results of this study too greatly.

## Conclusion

In asymptomatic or mildly symptomatic patients with primary moderate or severe MR, an increase in the LAVI at rest indicates the requirement for ESE, even without LV dilatation, which is an indication for surgery in current guidelines. ESE can help to identify patients with MR who are candidates for early surgical intervention.

## Figures and Tables

**Table1:** Patients’ characteristics

Variables	Group I (n=11)	Group II (n=30)	P value
Age, years	65±12	54±14	0.042
Male, n (%)	6 (55)	20 (67)	0.475
Body surface area, m^2^	1.62±0.21	1.67±0.17	0.735
Heart rate, beats/min	75±11	72±12	0.361
Systolic blood pressure, mmHg	140±27	135±21	0.546
Diastolic blood pressure, mmHg	78±23	76±13	0.768
History			
Hypertension, n (%)	9 (82)	12 (40)	0.018
Dyslipidemia, n (%)	6 (55)	11 (37)	0.303
Diabetes mellitus, n (%)	0 (0)	3 (10)	0.276
Medications			
ACE-I/ARB, n (%)	5 (45)	10 (33)	0.475
Beta-blocker, n (%)	1 (9)	2 (7)	0.792
Calcium channel blocker, n (%)	1 (9)	5 (17)	0.543
Diuretic, n (%)	4 (36)	3 (10)	0.047
NT-proBNP, pg/mL	351±278	125±163	0.002
Chest X-ray CTR (%)	53±5	49±6	0.060
Exercise heart rate, beats/min	134±23	128±15	0.480
Exercise systolic blood pressure, mmHg	199±29	191±23	0.659
Exercise diastolic blood pressure, mmHg	92±21	86±16	0.257
Maximal exercise workload, W	68±23	87±31	0.069

Numeric values are expressed as the mean±standard deviation. ACE-I, angiotensin-converting enzyme inhibitor; ARB, angiotensin II receptor blocker; NT-proBNP, N-terminal pro-B-type natriuretic peptide; CTR, cardiothoracic ratio.

**Table2:** Echocardiographic parameters

Variables	Group I (n=11)	Group II (n=30)	P value
Rest			
LVEDDI, mm/m^2^	32±4	30±3	0.035
LVESDI, mm/m^2^	19±3	18±3	0.180
LVEDVI, mL/m^2^	64±12	57±10	0.085
LVESVI, mL/m^2^	23±4	21±5	0.205
LVEF, %	64±4	64±3	0.829
LAVI, mL/m^2^	45±14	30±11	0.008
Peak E wave velocity, cm/sec	125±45	101±24	0.050
TRPG, mmHg	28±9	19±8	0.018
Peak exercise			
LVEDDI, mm/m^2^	30±11	28±6	0.531
LVESDI, mm/m^2^	18±6	17±5	0.553
LVEDVI, mL/m^2^	59±12	52±13	0.144
LVESVI, mL/m^2^	20±5	17±5	0.108
LVEF, %	66±5	68±5	0.466
LAVI, mL/m^2^	49±12	33±14	0.008
TRPG, mmHg	63±6	35±15	0.994

Numeric values are expressed as the mean±standard deviation. LVEDDI, left ventricular end-diastolic diameter index; LVESDI, left ventricular end-systolic diameter index; LVEDVI, left ventricular end-diastolic volume index; LVESVI, left ventricular end-systolic volume index; LVEF, left ventricular ejection fraction; LAVI, left atrial volume index; TRPG, tricuspid regurgitation pressure gradient.

**Table3:** Multivariate analysis

	HR (95% CI)	P value
Peak E wave velocity	1.021 (0.990–1.054)	0.190
LVEDDI	1.145 (0.851–1.542)	0.372
LAVI	1.081 (1.009–1.158)	0.028

HR, hazard ratio; CI, confidence interval; LVEDDI, left ventricular end-diastolic diameter index; LAVI, left atrial volume index.
